# Dendritic cells in reflectance confocal microscopy are a clue for early melanoma diagnosis in extrafacial flat pigmented melanocytic lesions

**DOI:** 10.1111/exd.14553

**Published:** 2022-03-04

**Authors:** Laura Guiducci, Shaniko Kaleci, Johanna Chester, Caterina Longo, Silvana Ciardo, Francesca Farnetani, Giovanni Pellacani

**Affiliations:** ^1^ Department of Dermatology University of Modena and Reggio Emilia Modena Italy; ^2^ Azienda Unità Sanitaria Locale – IRCCS di Reggio Emilia Centro Oncologico ad Alta Tecnologia Diagnostica‐Dermatologia Reggio Emilia Italy; ^3^ Dermatology Clinic Department of Clinical Internal, Anesthesiological and Cardiovascular Sciences Sapienza University of Rome Rome Italy

**Keywords:** dendritic cells, extrafacial pigmented lesions, melanoma, nevi, reflectance confocal microscopy

## Abstract

Differential diagnosis of extrafacial flat pigmented lesions with dermoscopic reticular and/or homogeneous pattern is challenging. Dendritic cells upon reflectance confocal microscopy (RCM) still represent a pitfall. This study aims to determine the role of dendritic cells upon RCM in the epidermis and dermo‐epidermal junction (DEJ), together with common RCM features for melanoma and nevi, in dermoscopically equivocal extrafacial flat pigmented lesions. A retrospective evaluation of RCM images of melanocytic extrafacial flat pigmented lesions with reticular and/or homogeneous dermoscopic pattern and with histopathological diagnosis, was performed. A multivariate model of RCM features was used to obtain a score of independent risk factors. A total of 698 lesions were included. Increasing patient age, epidermal dendritic cells, many dendritic cells in the DEJ (>30%) and many (>5/mm^2^) round atypical cells were independent risk factors for melanoma. Edged papillae and melanophages were indicative of nevus. A score based on these features was developed to assist in melanoma differential diagnosis. The RCM observation of abundant (>30%) dendritic cells in the DEJ is highly suggestive of malignity. This independent risk factor should also be considered for improved differential diagnosis of extrafacial melanoma.

## 1 INTRODUCTION

Dermoscopy improves skin cancer detection sensitivity and reduces benign/malignant ratio of excised lesions.^1^, ^2^ Reflectance confocal microscopy (RCM) can further improve skin lesion diagnostic accuracy with in vivo visualization of the epidermis and superficial dermis in real time correlating well with dermoscopic and histopathologic findings.^3^

However, RCM is limited by the lack of nuclear staining, limited imaging depth, difficulty in visualizing nodular lesions and distinguishing dendritic melanocytes in pagetoid pattern from Langerhans cells, which can occasionally simulate pagetoid spread.^4^ Therefore, RCM evaluators need to consider all cellular and architectural lesion characteristics to improve agreement with final histopathologic diagnosis.^5^

Common differential diagnostic RCM features have been previously published. Melanoma is typically observed with a disarranged honeycomb pattern and bright‐nucleated cells in a pagetoid spread in the suprabasal layers and non‐edged dermal papillae, with atypical melanocytes and/or foci with loss of the dermal papillae at the DEJ.^4^, ^6^, ^7^ The presence of atypical dendritic cells, in particular infiltrating the hair follicle (folliculotropism) at the DEJ, has been proven to be a highly specific RCM pattern for facial lentigo maligna (LM)/lentigo maligna melanoma (LMM) diagnosis.^8^, ^9^, ^10^

For extrafacial lesions, melanoma was described as characterized by pagetoid infiltration of round cells and/or dendritic cells and the focal proliferation of dendritic pagetoid cells in the epidermis in selected lesions on chronically sun‐damaged skin,^11^ and the presence of junctional cytological atypia (where roundish and dendritic cells were considered together) was the strongest RCM predictive factor for in situ melanoma.^12^

Differential diagnosis between atypical melanocytes and Langerhans cells with cell morphology visualized at RCM alone cannot be achieved. However, Segura et al. identified dendritic cells in pigmented basal cell carcinomas and, with the aid of immunohistochemistry, concluded that dendritic cells in the tumoral basaloid nests correspond to melanocytes, whereas dendritic cells in the epidermis correspond to Langerhans cells.^13^ Therefore, we hypothesize that cell distribution, depth and architecture may assist in RCM differential diagnosis.

The aim of the current study was to correlate common RCM patterns of the epidermis and DEJ, (dendritic and roundish cells considered separately) and their distribution, depth and architecture in extrafacial melanocytic lesions with common dermoscopic presentation (i.e. reticular and/or homogeneous pattern) and without dermoscopic melanoma specific clues, diagnosed nevi or melanoma. The combination of features indicative of melanoma and the subsequent development of a score to assist in differential diagnosis is the secondary aim of the study.

## 2 METHODS

### 2.1 Study data set

We performed a retrospective analysis of consecutive extrafacial lesion images with histopathological diagnosis, maintained in a dedicated database at the Dermatology Department, University of Modena and Reggio Emilia, acquired between October 2015 and March 2020. Further study inclusion specified flat lesions, with homogeneous and/or reticular pattern at dermoscopy,^14^ without dermoscopic criteria of growth and malignancy (streaks, rim of peripheral globules or dots, starbust pattern, pseudopods, irregular globules or dots),^15^, ^16^, ^17^ regression >50% (blue‐white structures such as blue‐white veil, shiny white structures and grey dot granules),^18^ dermoscopic eccentric blotches and multicolour pattern (≥3 colors).^15^, ^16^, ^17^

### 2.2 Image acquisition and analysis

Standardized polarized dermoscopic clinical images were obtained with DermLite Photo (3Gen) mounted on a Canon G16 camera. In vivo RCM images (Vivascope 1500; Mavig GmbH) were captured according to a standardized procedure previously described.^19^

RCM mosaic images were obtained at the suprabasal epidermis (spinous and granular layers), DEJ and papillary dermis. All three RCM mosaic images/lesion were evaluated by a single clinician for the presence of RCM parameters, published previously, see Table S1.^6^, ^19^, ^20^, ^21^, ^22^, ^23^ Percentage of presence of selected features were calculated by counting the squares of the mosaic block when observed. The reader was blinded to final histopathological diagnosis.

Biopsy specimens were analysed by a dermatopathologist following fixation in formaldehyde, embedding in paraffin, sectioning and staining with haematoxylin‐eosin.

### 2.3 Confocal dendritic cells‐index: A predictive score for melanoma diagnosis

Based on prognostic factors identified with logistic regression, a score for melanoma diagnosis probability was devised. Briefly, each prognostic variable is assigned a score (0–10). For simplicity, age ranges were created and dendritic cells at the DEJ combined both absent (0%) and <10%. Total confocal dendritic cells‐index (CDC–I) scores (0–52) correspond to melanoma diagnosis probabilities.

### 2.4 Statistical analysis

Statistical analysis was performed using STATA^®^ (v14; StataCorp. 2015. Stata Statistical Software: Release 14: StataCorp LP.). Continuous variables (patients [N], mean, standard deviation [SD]) were compared using Unpaired Student's t (2 groups) or Anova (>2 groups). Categorical variables (frequency [N, %]) were compared using Pearson's chi‐squared test.

Logistic regression model (stepwise forward selection) was used for association between parameters and to identify prognostic factors. Intercept‐only model was fitted and individual score statistics were evaluated (*p* < 0.05), removing insignificant variables before adding variables. Data were expressed as odds ratio (OR), 95% confidence interval (CI). *p* < 0.05 was considered statistically significant. A nomogram for predicting melanoma probability (including univariate and multivariate logistic regression analyses) screened for fit predictors. Nomogram predictability was assessed with area under the curve (AUC), by receiver operating characteristic (ROC) analysis.

## 3 RESULTS

A total of 698 extrafacial, flat, melanocytic lesions (621 patients) met inclusion criteria and were enrolled. Most patients were male (56.4%) and the majority of lesions were located on the trunk (72.9%). Histopathological diagnoses revealed predominantly compound nevi (34%), followed by junctional nevi (31%), melanoma in situ (23%) and invasive melanomas (12%). The mean Breslow index for invasive melanomas was 0.36 mm ± 0.14 (range 0.1–0.8).

Retrospective RCM image analysis revealed dendritic cells in the epidermis in over two thirds of the lesions. According to histopathological diagnosis, dendritic cells were observed in over 80% of the in situ and invasive melanomas, whilst they were less frequently observed in junctional and compound nevi. Almost all in situ and invasive melanomas had atypia in the epidermis, and almost all lesions with a regular epidermis were associated with compound and junctional nevi diagnoses (*p* < 0.001), see Table 1.

**TABLE 1:** Frequency of reflectance confocal microscopy features observed at the epidermis and dermo‐epidermal junction (DEJ) layers of included lesions, correlated according to final histopathological diagnosis

Reflectance Confocal microscopy features		Total *n* (%)	In situ melanomas *n* (%)	Invasive melanomas *n* (%)	Compound nevi *n* (%)	Junctional nevi *n* (%)	p‐value
		698 (100)	160 (23.0)	84 (12.0)	237 (34.0)	217 (31.0)	
Epidermis							
Pattern	Regular	55 (7.9)	1 (0.6)	1 (1.2)	34 (14.3)	19 (8.8)	<0.001
	Irregular	150 (21.5)	16 (10.0)	9 (10.7)	80 (33.8)	45 (20.7)	
Dendritic cells/tangled lines	Present	493 (70.6)	143 (89.4)	74 (88.1)	123 (51.9)	153 (70.5)	
Dermal‐epidermal junction							
Edged papillae	Absent	19 (2.7)	8 (5.0)	7 (8.3)	1 (0.4)	3 (1.4)	<0.001
	Present	679 (97.3)	152 (95.0)	77 (91.7)	236 (99.6)	214 (98.6)	
Ring pattern	0%	102 (14.6)	34 (21.3)	20 (23.8)	27 (11.4)	21 (9.7)	0.014
	<10%	107 (15.3)	24 (15.0)	14 (16.7)	37 (15.6)	32 (14.7)	
	10%–30%	122 (17.5)	32 (20.0)	14 (16.7)	38 (16.0)	38 (17.5)	
	30%–50%	76 (10.9)	20 (12.5)	8 (9.5)	25 (10.5)	23 (10.6)	
	>50%	291 (41.7)	50 (31.3)	28 (33.3)	110 (46.4)	103 (47.5)	
Mesh pattern	0%	226 (32.4)	51 (31.9)	34 (40.5)	72 (30.4)	69 (31.8)	0.260
	<10%	68 (9.7)	16 (10.0)	8 (9.5)	17 (7.2)	27 (12.4)	
	10%–30%	106 (15.2)	16 (10.0)	9 (10.7)	45 (19.0)	36 (16.6)	
	30%–50%	83 (11.9)	23 (14.4)	11 (13.1)	27 (11.4)	22 (10.1)	
	>50%	215 (30.8)	54 (33.8)	22 (26.2)	76 (32.1)	63 (29.0)	
Aspecific pattern	0%	229 (32.8)	29 (18.1)	7 (8.3)	105 (44.3)	88 (40.6)	<0.001
	<10%	109 (15.6)	27 (16.9)	11 (13.1)	32 (13.5)	39 (18.0)	
	10%–30%	166 (23.8)	46 (28.8)	21 (25.0)	57 (24.1)	42 (19.4)	
	30%–50%	80 (11.5)	22 (13.8)	14 (16.7)	22 (9.3)	22 (10.1)	
	>50%	114 (16.3)	36 (22.5)	31 (36.9)	21 (8.9)	26 (12.0)	
Non edged papillae	0%	440 (63.0)	89 (55.6)	44 (52.4)	154 (65.0)	153 (70.5)	0.057
	<10%	53 (7.6)	14 (8.8)	7 (8.3)	18 (7.6)	14 (6.5)	
	10%–30%	90 (12.9)	24 (15.0)	12 (14.3)	31 (13.1)	23 (10.6)	
	30%–50%	61 (8.7)	18 (11.3)	9 (10.7)	23 (9.7)	11 (5.1)	
	>50%	54 (7.7)	15 (9.4)	12 (14.3)	11 (4.6)	16 (7.4)	
Flattening	0%	648 (92.8)	147 (91.9)	63 (75.0)*	228 (96.2)	210 (96.8)	<0.001
	<10%	8 (1.1)	1 (0.6)	4 (4.8)	0 (0.0)	3 (1.4)	
	10%–30%	15 (2.1)	2 (1.3)	8 (9.5)	5 (2.1)	0 (0.0)	
	30‐50	8 (1.1)	2 (1.3)	2 (2.4)	3 (1.3)	1 (0.5)	
	>50%	18 (2.6)	8 (5.0)	6 (7.1)	1 (0.4)	3 (1.4)	
Dendritic cells/tangled lines	0%	96 (13.8)	6 (3.8)	3 (3.6)	63 (26.6)	24 (11.1)	<0.001
	<10%	89 (12.8)	9 (5.6)	2 (2.4)	35 (14.8)	43 (19.8)	
	10%–30%	211 (30.2)	38 (23.8)	15 (17.9)	80 (33.8)	78 (35.9)	
	30%–50%	185 (26.5)	60 (37.5)	35 (41.7)	46 (19.4)	44 (20.3)	
	>50%	117 (16.8)	47 (29.4)	29 (34.5)	13 (5.5)	28 (12.9)	
Density of dendritic cells	Absent	96 (13.8)	6 (3.8)	3 (3.6)	63 (26.6)	24 (11.1)	<0.001
	Scattered	104 (14.9)	10 (6.3)	4 (4.8)	46 (19.4)	44 (20.3)	
	Intermediate	258 (37.0)	59 (36.9)	19 (22.6)	81 (34.2)	99 (45.6)	
	Dense	240 (34.4)	85 (53.1)	58 (69.0)	47 (19.8)	50 (23.0)	
Round and/or oval atypical cells	Absent	504 (72.2)	92 (57.5)	32 (38.1)**	189 (79.7)	191 (88.0)	<0.001
	<5/mm²	128 (18.3)	52 (32.5)	21 (25.0)	35 (14.8)	20 (9.2)	
	5‐10/mm²	50 (7.2)	13 (8.1)	23 (27.4)	9 (3.8)	5 (2.3)	
	>10/mm²	16 (2.3)	3 (1.9)	8 (9.5)	4 (1.7)	1 (0.5)	
Melanophages	Absent	644 (92.3)	153 (95.6)	77 (91.7)	212 (89.5)	202 (93.1)	0.144
	Abundant	54 (7.7)	7 (4.4)	7 (8.3)	25 (10.5)	15 (6.9)	

*p value <0.002 in situ melanoma vs invasive melanoma; **p value <0.001 in situ melanoma vs invasive melanoma.

At the DEJ, edged papillae were present in the majority of the lesions. The few cases of absent edged papillae were mainly associated with in situ (*n* = 8) and invasive melanomas (*n* = 7), compared with compound and junctional nevi (*p* < 0.001). Ring pattern was mostly associated with benign lesions; >50% ring pattern was observed in almost half of the compound and junctional nevi, and the absence of the ring pattern was mainly observed in in situ and invasive melanomas, *p* = 0.014. An inverse observation was noted for aspecific pattern, which was mostly associated with in situ and invasive melanomas (<0.001).

Overall, non‐edged papillae were observed in only 37% of the included lesions, and there were no significant differences observed between the diagnostic groups (*p* = 0.057). Flattening of the DEJ, was rarely observed (almost 7% of the lesions), with its presence mostly associated with invasive melanomas (*p* < 0.001).

The presence and frequency of dendritic cells at the DEJ was distributed relatively evenly, but distribution on >30% of the lesion was mostly associated with in situ and invasive melanomas. The higher frequency of dendritic cells in in situ and invasive melanomas was also associated with a higher density (>30% of the lesion area), compared with compound and junctional nevi where most lesions had absent, scarse or intermediate (<30%) dendritic cell density (*p* < 0.001).

Overall, most lesions did not have atypical round and /or oval cells (72.2%), and any eventual presence and higher density was mostly associated with in situ or invasive melanomas (*p* < 0.001).

Only 54 lesions (7.7%) presented abundant melanophages and were insignificantly more frequently observed in compound nevi.

Features suggestive of an invasive compared with in situ melanoma diagnosis included flattening (*p* = 0.002) and the presence of round cells in >5 mm^2^ (*p* < 0.001) of the DEJ.

At multivariable analysis, increasing patient age (proportionally, OR = 1.04, CI 1.03‐1.06, *p* < 0.0001), dendritic cells in the epidermis (OR = 7.54, CI 1.7–33.4, *p* < 0.008), dendritic cells at the DEJ >30% of lesion area (OR = 5.15, CI 2.79–9.53, *p* < 0.0001 if 30%–50% and OR = 7.12, CI 5.55–14.28, *p* < 0.0001 if >50%) and increasing presence of round and/or oval cells (OR = 4.20, CI 2.56–6.89, *p* < 0.0001 if <5/mm², OR = 6.81, CI 3.13–14.80, *p* < 0.0001 if 5–10/mm², OR = 5.70, CI 1.62–20.15, *p* < 0.007 if >10/mm², respectively) increased the likelihood of a melanoma diagnosis. Conversely, edged papillae (OR = 0.29, CI 0.08–0.96, *p* < 0.043) and melanophages (OR = 0.24, CI 0.11–0.53, *p* < 0.0001) decreased the risk of melanoma diagnosis, see Table 2.

**TABLE 2:** Multivariate logistic regression model risk for a melanoma diagnosis, including all statistically significant variables identified in univariate analysis (*p* < 0.05)

	OR (95% CI)	*p*‐value
Age, years	1.04 (1.03‐1.06)	<0.001
Age categories		
≤50	Ref.	
50–65	1.73 (1.09–2.74)	0.190
65–75	3.04 (1.77–5.19)	<0.001
>75	7.51 (3.67–15.39)	<0.001
Epidermis		
Regular	Ref.	
Irregular pattern	4.55 (0.97–21.30)	0.054
Presence of dendritic cells	7.54 (1.70–33.40)	0.008
Edged papillae	0.29 (0.08–0.96)	0.043
Dendritic cells at dermo–epidermal junction		
0%–10%	Ref.	
10%–30%	1.91 (1.03–3.55)	0.040
30%–50%	5.15 (2.79–9.53)	<0.001
>50%	7.12 (5.55–14.12)	<0.001
Round and/or oval atypical cells:		
Absent	Ref.	
<5/mm²	4.20 (2.56–6.89)	<0.001
5–10/mm²	6.81 (3.13–14.80)	<0.001
>10/mm²	5.70 (1.62–20.15)	0.007
Abundant melanophages	0.24 (0.11–0.53)	<0.001

Abbreviations: CI, confidence interval; OR, odds ratio.

### 3.1 Confocal dendritic cells—index

**FIGURE 1:**
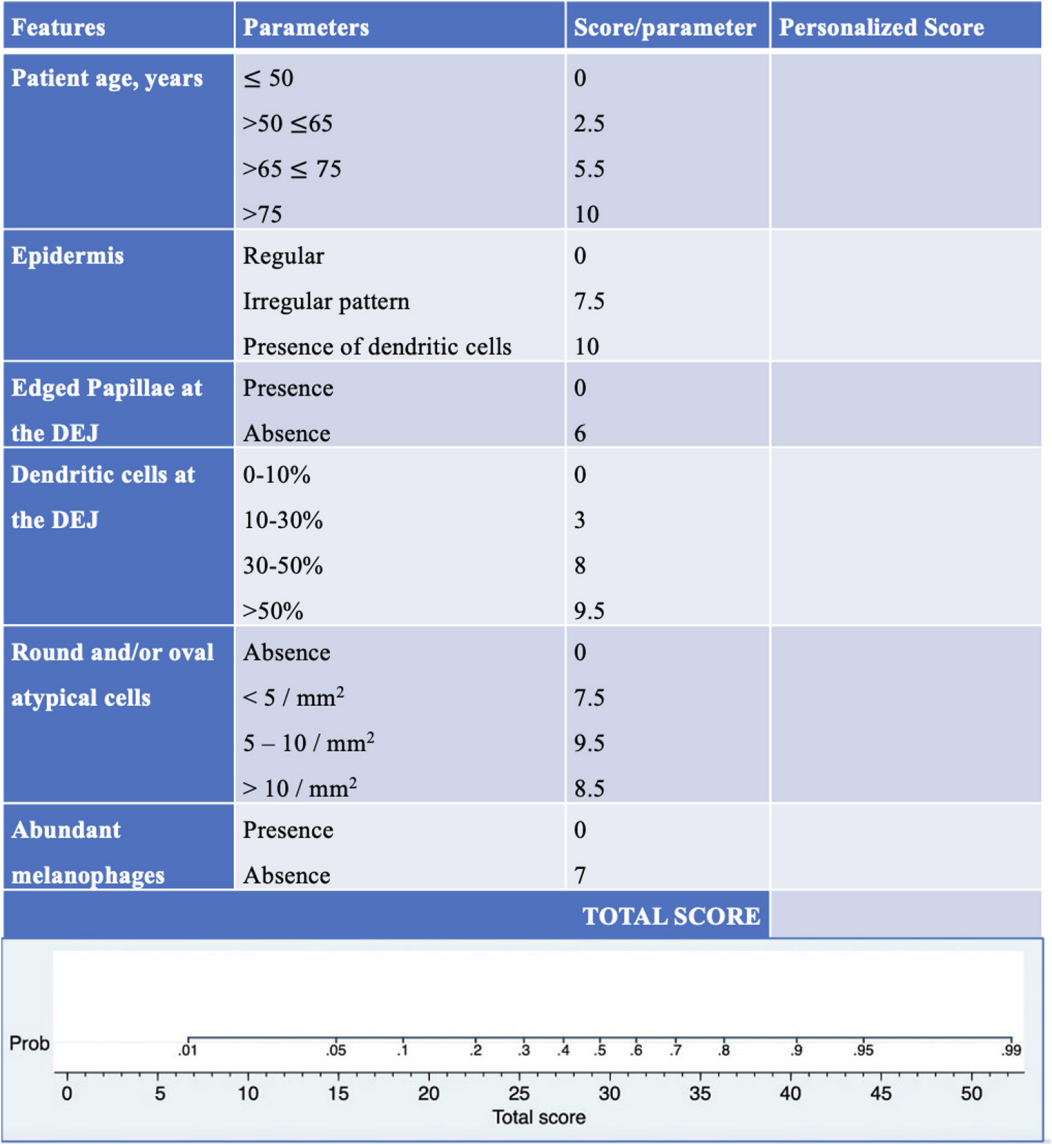
Confocal dendritic cell—index (CDC‐I). Worksheet for the application of the Proposed Predictive Score for melanoma diagnosis

**FIGURE 2:**
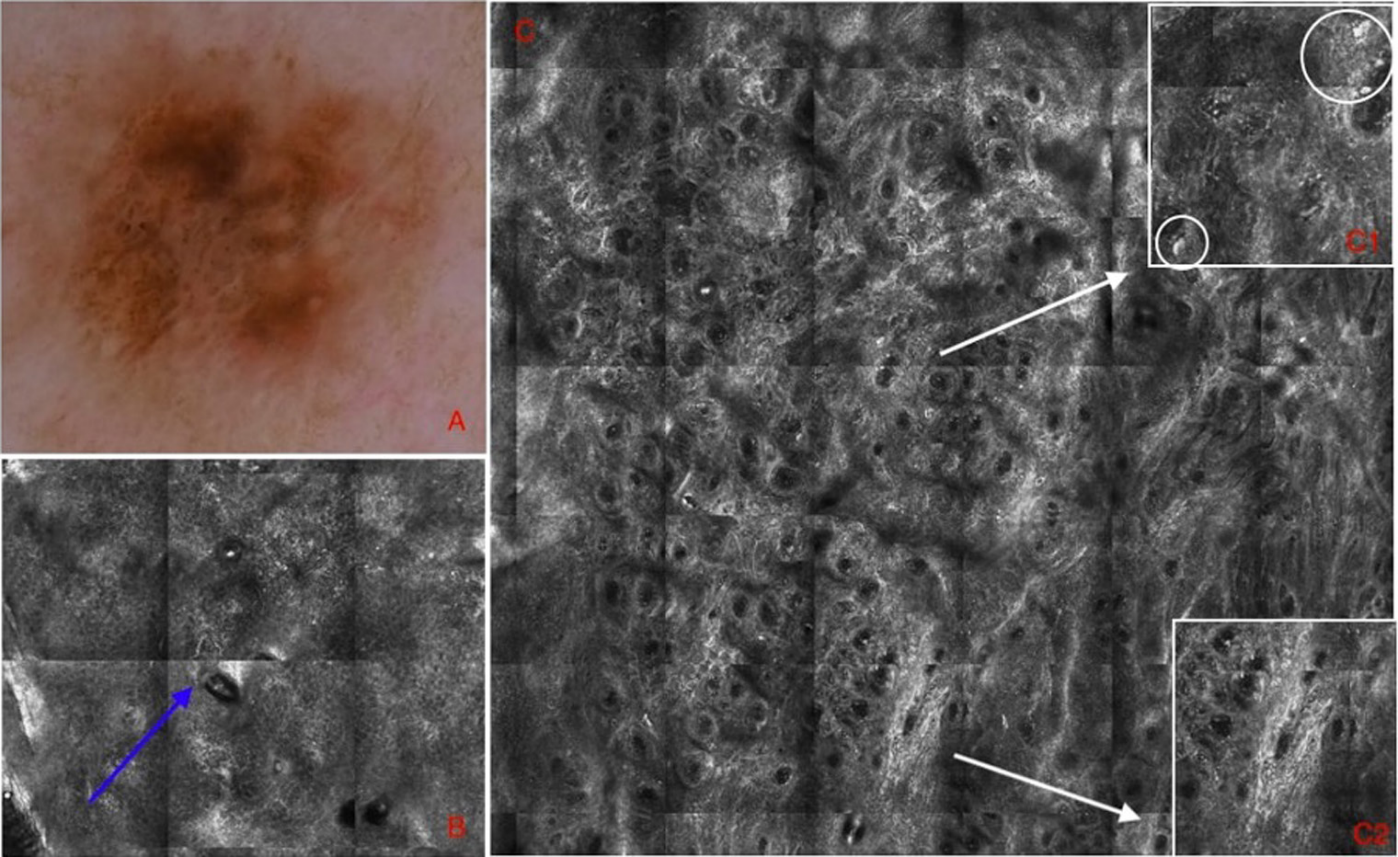
*In situ melanoma* Lesion 1. (A) Dermoscopy image acquired at baseline. The lesion was located on the right shoulder of a 48‐year‐old female patient (score = 0) (B) Reflectance confocal microscopy (RCM) highlighted the presence of dendritic cells in the epidermis (blue arrow; score = 10), (C) the absence of edged papillae and melanophages in the dermo‐epidermal junction (DEJ) (score = 6 + 7, respectively), (C1) and the presence of >5 mm2 atypical cells (white circle; score 7.5) and (C2) dendritic cells in >50% of the lesion area (score = 9.5). Lesion total score was 40, resulting in 90% probability for melanoma diagnosis

**FIGURE 3:**
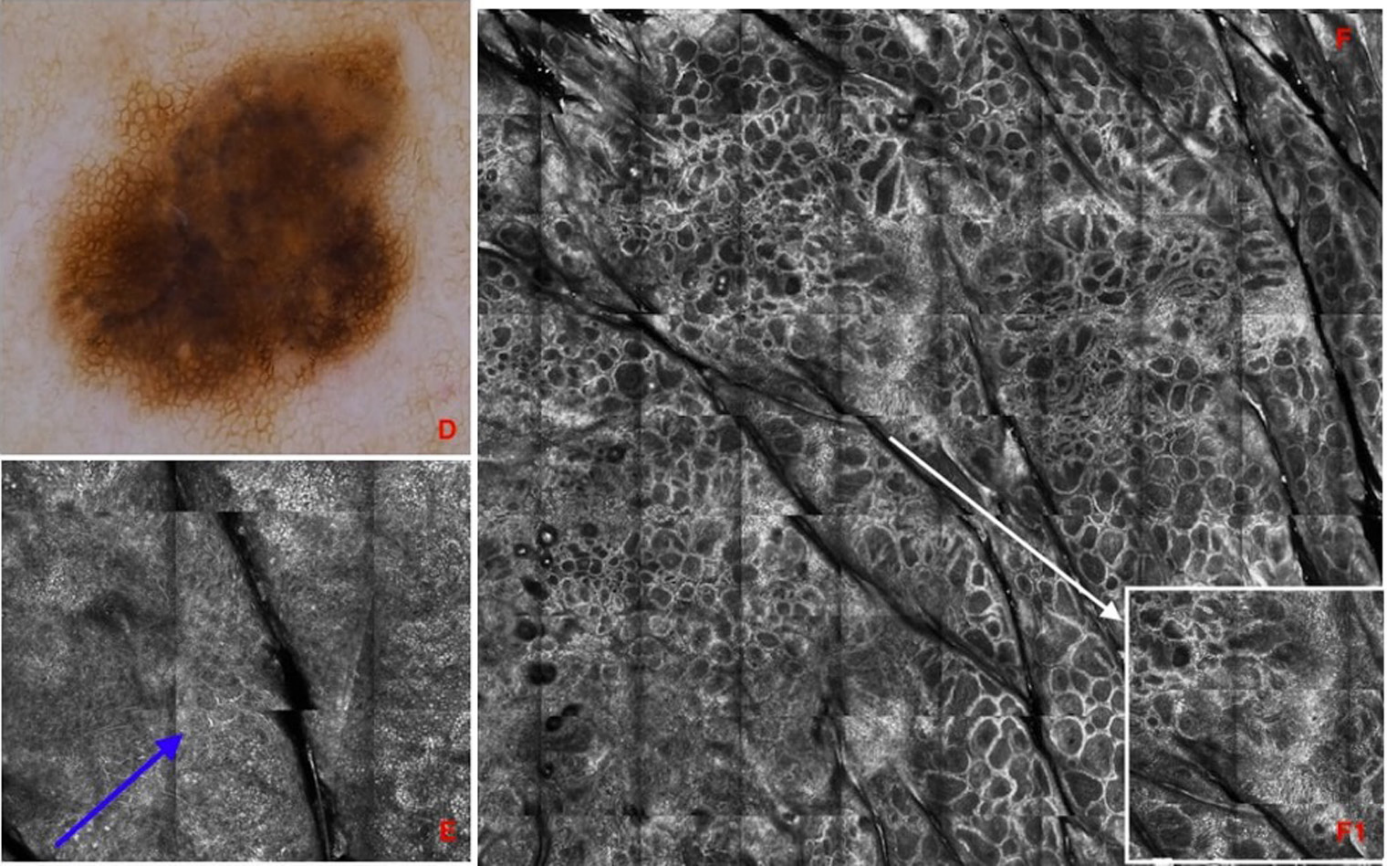
*Junctional Nevus* Lesion 2. (D) Dermoscopy image acquired at baseline. The lesion was located on the right shoulder of a 72‐year‐old male (score 5.5). (E) Reflectance confocal microscopy (RCM) highlighted the presence of dendritic cells in the epidermis (blue arrow; score = 10), (F) edged papillae in a ring pattern, dendritic cells in 10‐30% of the lesion area (F1) and in the center of the lesion, (F) absence of melanophages and round and/or oval atypical cells in the dermo‐epidermal junction (DEJ) (score =0 + 3 + 7 + 0 respectively). Lesion total score was 25.5, resulting in 30% probability for melanoma diagnosis

Confocal dendritic cells—index (CDC–I), including predictive variables identified with regression analysis, was developed from the Nomogram analysis. A worksheet (Figure 1) enables clinicians to calculate a personalized CDC‐I score, corresponding to a probability of a melanoma diagnosis demonstrates the application of CDC‐I in two lesion examples (Figures 2,3). The CDC‐I predictive accuracy was high (AUC = 0.84).

## 4 DISCUSSION

This large study confirms common RCM features for differential diagnosis between melanomas and nevi for extrafacial lesions^4^, ^6^ and more recent findings associating dendritic cells in the epidermis of chronic sun‐damaged skin with melanoma.^11^ The presence of abundant dendritic cells in the DEJ is a differential diagnostic feature.

Pellacani et al., hypothesized that dendritic cells could be the RCM hallmark of slow‐growing melanomas.^7^ In facial LM studies, tangled lines are reported as a specific feature,^8^, ^9^, ^24^, ^25^, ^26^ and in extrafacial skin melanomas, the predominant feature of melanocytes was the dendritic cell‐type morphology.^11^ However, many studies have shown bright epidermal dendritic cells in a considerable number of melanotic lesions, representing a possible diagnostic pitfall in melanoma differentiation. Cells with long/thin dendritic‐like branches have been found to have no certain correlation with histopathology, although the shape may suggest its correspondence with Langerhans cells.^5^, ^6^

Dendritic cells observed with RCM cannot be differentiated from Langerhans cells (observed clearly in histopathology) because the same dendritic morphology is shared. However, this study reports that dendritic cells deeper at the DEJ is sensitive for melanoma diagnosis, only when present in more than 30% of the lesion. These data suggest that dendritic cells are mostly correlated with single cell melanocytic proliferation. When dendritic cells are abundant and continuous, the diagnosis is more likely melanoma, whereas fewer dendritic cells seem to indicate benign lentiginous proliferations. Dendritic cells observed in the epidermis is also confirmed in this study to be sensitive for melanoma but not specific, as they are also present in the epidermis of nevi (particularly junctional nevi), whereas atypical round or oval cells at the DEJ are sensitive for malignancy, independent of extent.

Atypical round or oval cells at the DEJ, on the contrary, are sensitive for malignancy, independent of extent. Further studies with RCM monitoring of the evolution of lesions with a limited extent of dendritic cells may better clarify the role of this entity in the pathogenetic development of slow‐growing melanomas. Edged papillae and abundant melanophages are indicative of nevi, as previously demonstrated by Borsari et al.^12^ These RCM features, along with increasing age, had a good sensitivity and specificity for differential diagnosis (AUC = 0.84). In our study, other RCM features, such as aspecific pattern, non‐edged papillae and flattening of the DEJ, significantly correlated with melanomas, but with low specificity.

Differential diagnoses between invasive and in situ extrafacial melanoma are assisted by flattening and abundant round cells of the DEJ. Atypical cells in the DEJ were confirmed as the strongest RCM predictive factor at multivariable analysis for an in situ melanoma diagnosis compared to nevi in a series of 333 extrafacial lesions^12^ and as a was identified as the main feature for melanomas with a Breslow index between 0.01 and 1.0 mm.^7^

The current study is limited by a retrospective design, image evaluation by a single clinician only, the lack of inter‐personal evaluator agreement and lesion selection bias, excluding unequivocal nevi. Analysis did not include assessment of the features according to melanoma invasiveness, but could be considered in future studies. Further, the lack of immunohistochemistry analysis does not enable a clear distinction in this study between melanocytes and Langerhans' cells.^27^

The authors recommend lesion excision if:

- Dendritic cells/ tangled lines are present in >30% of lesion surface, or
- Edged papillae are absent
- CDC—I score is >15 (50% probability of a melanoma diagnosis).

Despite lacking correlation between RCM dendritic morphology and benign or malignant melanocytes or Langerhans cells upon histology, the extent (>30%) and density of dendritic cells at the DEJ seem to be indicative of melanocytic proliferation, and therefore indicative of melanoma. Our study confirms that the abundant presence of round cells in the DEJ assist in identifying invasive melanoma. The reproducibility of the CDC—I score requires validation in other samples prior to being applied to clinical practice.

## CONFLICT OF INTEREST

None to declare.

## AUTHOR CONTRIBUTIONS

All authors have read and approved the final manuscript. LG performed the research, designed the research study, contributed essential reagents or tools and analysed the data. SK designed the research study, contributed essential reagents or tools, analysed the data and wrote the paper. JC designed the research study, analysed the data and wrote the paper. CL contributed essential reagents or tools. SC performed the research and contributed essential reagents or tools. FF performed the research and analysed the data. GP designed the research study, analysed the data and wrote the paper.

## ETHICS STATEMENT

This retrospective study was approved by the local ethics committee (Prot. AOU 0008852/20 of 25/03/2020.).

## PATIENT CONSENT STATEMENT

All patients consented to the storage and further analysis of clinical and lesion data for research purposes.

## PERMISSION TO REPRODUCE MATERIAL FROM OTHER SOURCES

Not applicable.

## Supporting information


**Table S1**. Definition of evaluated reflectance confocal microscopy (RCM) parameters.

## Data Availability

The data that support the findings of this study are available from the corresponding author upon reasonable request.
