# Supplementary material for: Dendritic cells in reflectance confocal microscopy are a clue for early melanoma diagnosis in extrafacial flat pigmented melanocytic lesions
Source: Exp Dermatol. 2022 Mar 4;31(7):1048–55. doi: 10.1111/exd.14553 (PMC9542116; doi:10.1111/exd.14553)
Supplement: Supplementary file 1 — Table S1. Definition of evaluated reflectance confocal microscopy (RCM) parameters. [file EXD-31-1048-s001.docx]

Supplementary Table 1: Definition of evaluated reflectance confocal microscopy (RCM) parameters

| **RCM parameters** | **Definition** |
| --- | --- |
| **EPIDERMIS** | |
| Regular | The suprabasal epidermis is characterized by a regular honeycomb pattern (thin bright polygonal outlines that are even in thickness, brightness, size and shape) or regular cobblestone pattern (closely set bright round cells, even in size, brightness, with uniform spacing, separated by a less refractive polygonal outlines) **(20, 21)** |
| Irregular | The suprabasal epidermis is characterized by an irregular honeycomb pattern (polygonal outlines that vary in thickness and brightness of the lines, and the size and shape of the holes); broadened honeycomb pattern (the polygonal outlines are uniformly thickened and often brighter than normal); irregular cobblestone pattern (closely set bright round cells that vary in size and brightness and display disarray); or disarranged epidermis (lack of recognizable honeycomb or cobblestone patterns, often with unevenly distributed bright granular particles and cells) **(20, 21)** |
| Presence of dendritic cells / tangled lines | Presence of bright dendritic cells at suprabasal layers of the epidermis **(22).** Tangled lines are bright filaments where the bodies of the cells are barely visible **(23).** |
| **DERMO-EPIDERMAL JUNCTION** | |
| Presence of edged papillae | Presence of dermal papillae demarcated by a rim of bright cells (pigmented basal keratinocytes and melanocytes). **(20)** |
| Presence and percentage of non edged papillae | Dermal papillae without a demarcated bright rim at the DEJ **(20)**. Measured in terms of quantitative presence in the lesion image, where 100% is considered the whole lesion: 0: 0%; 1: 0-10%, 2: 10-30%, 3: 30-50%, 4: >50% |
| Overall DEJ pattern and percentage of presence | Ring pattern: rims of small bright cells surrounding papillae (appearing as bright rings) sharply contrasting with the dark background;  Meshwork pattern: junctional thickenings, corresponding to enlargements of the interpapillary spaces formed by clusters of melanocytes;  Aspecific pattern: lack of one of the previous patterns, resulting in a non-specific architecture (undefined structures);  Flattening: flattened rete ridged due to dermal-epidermal junction disruption; **(20)**  Measured in terms of quantitative presence in the lesion image, where 100% is considered the whole lesion: 0: 0%; 1: 0-10%, 2: 10-30%, 3: 30-50%, 4: >50% |
| Presence and percentage of dendritic cells / tangled lines | Presence of bright dendritic cells at dermo-epidermal junction and/or of tangled lines. Dendrite is an elongated branching structure extending from the cell body **(20).** Tangled lines are bright filaments where the bodies of the cells are barely visible **(23).** Measured in terms of quantitative presence in the lesion image, where 100% is considered the whole lesion: 0: 0%; 1: 0-10%, 2: 10-30%, 3: 30-50%, 4: >50% |
| Density of dendritic cells | Quantification of density of dendritic cells according to cellularity: 1: scattered; 2: intermediate; 3: dense. |
| Presence and density of round and/or oval atypical cells | Large bright-nucleated cells (corresponding to atypical melanocytes). The density is evaluated according to number of cells /mm^2^: 0: absence; 1: <5 for mm²; 2: 5-10 for mm²; 3: >10 for mm². |
| Presence of melanophages | Melanophages are bright plump, oval or star-shaped cells with no visible nucleus, with ill-defined edges and they are smaller than round melanocytic cells **(24)**. Measured according to absence or abundance |
